# Piezophotocatalytic Activity of PVDF/Fe_3_O_4_ Nanofibers: Effect of Ultrasound Frequency and Light Source on the Decomposition of Methylene Blue

**DOI:** 10.1021/acsomega.5c01092

**Published:** 2025-05-29

**Authors:** Alina Rabadanova, Daud Selimov, Rashid R. Gulakhmedov, Asiyat G. Magomedova, Kipkurui Ronoh, Klára Částková, Dinara Sobola, Pavel Kaspar, Abdulatip Shuaibov, Magomed G. Abdurakhmanov, Murtazali K. Rabadanov, Shikhgasan M. Ramazanov, Farid Orudzhev

**Affiliations:** a Smart Materials Laboratory, 64913 Dagestan State University, M. Gadzhiev 43a., Makhachkala, Respublika Dagestan 367000, Russian Federation; b Amirkhanov Institute of Physics of Dagestan Federal Research Center, Russian Academy of Sciences, Ulitsa Yaragskogo 94, Makhachkala, Respublika Dagestan 367003, Russian Federation; c Faculty of Electrical Engineering and Communication, 48274 Brno University of Technology, Brno, South Moravian Region 61600, Czech Republic; d CEITEC − Central European Institute of Technology, 48274 Brno University of Technology, Purkynova 656/123, Brno, 612 00, Czech Republic; e Institute of Scientific Instruments, Czech Academy of Sciences, Brno, South Moravian Region 612 00, Czech Republic

## Abstract

This study investigates the piezophotocatalytic (PPhC) performance of electrospun nanofibrous membranes composed of polyvinylidene fluoride (PVDF) and magnetite (Fe_3_O_4_) nanoparticles. The composite membranes were synthesized via electrospinning, with optimized parameters to promote β-phase crystallinity and uniform fiber morphology. Structural and phase analyses by SEM, FTIR, Raman, and XPS confirmed the predominance of the electroactive β-phase (99.8%) in the composite, as well as strong interfacial interaction between Fe_3_O_4_ and the PVDF matrix. The composites exhibited significantly enhanced surface hydrophilicity and piezoelectric response compared to pristine PVDF. The piezoelectric potential generation was confirmed using a flexible piezoelectric nanogenerator (PENG), where a 3 × 1 cm membrane generated output voltages up to ∼2 V under periodic mechanical deformation at 4 Hz. Photocatalytic and piezophotocatalytic degradation of methylene blue (MB) was carried out under UV and visible light at varying ultrasonic frequencies. Maximum PPhC efficiency was achieved at 40 kHz, with 93% dye degradation in 60 min and a reaction rate constant exceeding the sum of photocatalysis and piezocatalysis by 13%, indicating a pronounced synergistic effect. Reactive oxygen species trapping and fluorescence spectroscopy confirmed ^•^OH as the dominant oxidant. H_2_O_2_ productivity under PPhC reached 1700 μmol·g^–1^·h^–1^ in pure water, with a light-to-chemical energy conversion efficiency of 0.26%. Additionally, experiments conducted under an alternating magnetic field (0.3 T, 1.3 Hz) demonstrated 50% MB degradation within 240 min, revealing the contribution of magnetoelectric coupling as an alternative catalytic activation mechanism. The results suggest that PVDF/Fe_3_O_4_ nanocomposites are highly promising for multifunctional catalytic applications, combining piezoelectric, photo-, and magnetoelectric activation for efficient water purification and green oxidant production.

## Graphical abstract

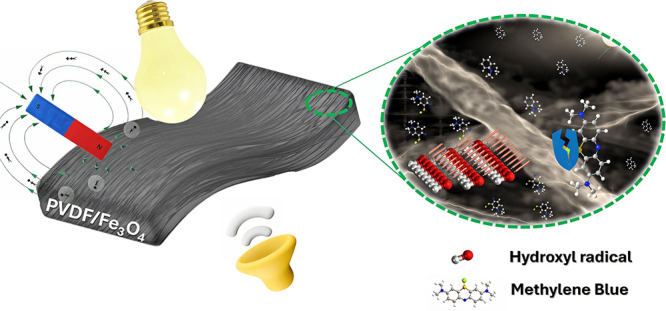

## 1 Introduction

Environmental pollution is one of the biggest problems facing the world today. Water problems are expected to worsen in the coming decades and water scarcity is expected to occur worldwide, even in regions that are currently considered water-rich. Solving these problems requires a tremendous amount of research to identify new reliable methods of cost-effective water purification, while minimizing the use of chemicals and environmental impact.^1^

Traditional water treatment methods include adsorption, filtration and membrane separation. However, for persistent organic pollutants, these approaches are often ineffective.^2^ In such cases, advanced oxidative processes (AOPs) are used. These technologies are based on the generation of reactive oxygen species (ROS) capable of breaking down complex organic compounds to simple molecules such as carbon dioxide and water through processes such as sonolysis, ozonation, UV, Fenton, etc.^3–5^

Photocatalysis based on the use of semiconductor materials occupies an important place among AOPs due to its efficiency and versatility. However, the key problem of photocatalysis - the high recombination rate of electrons and holes - limits its productivity. To overcome this barrier, strategies including doping, composite formation, creation of defect structures, and nanomorphology control have been developed.^6^ Although many methods have been proposed, finding a universal solution remains an open challenge.

In recent years, there has been a growing interest in using piezoelectric materials to enhance photocatalysis.^7^ The piezoelectric effect arises from mechanical deformation of a ferroelectric catalyst and causes generation of electric charge on its surface. This promotes the separation of photogenerated electron–hole pairs, increasing the amount of active oxygen species available for oxidation reactions. One of the most promising materials for piezostimulated photocatalysis is polyvinylidene fluoride (PVDF) due to its high piezoelectric activity, chemical stability and flexibility.^8–10^

PVDF exists in several phases, but the most important for piezoelectric applications is the β-phase, which is characterized by parallel orientation of its dipole moments. Achieving a high fraction of β-phase is possible by applying electrospinning techniques and modifying PVDF with nanoparticles such as Fe_3_O_4_ nanoparticles. It has been demonstrated that the incorporation of iron oxide (Fe_3_O_4_) nanoparticles promotes the formation of β-phase PVDF.^11,12^ Even more known is the active use of Fe_3_O_4_ in AOPs.^13,14^ So in the work of Zhou et al.^15^ it was shown that cavitation can lead to acceleration in combination with the UV/Fe_3_O_4_/oxalate system. In another work,^16^ the authors used Fe_3_O_4_ nanoparticles for the degradation of bisphenol A. The results showed that Fe_3_O_4_ nanoparticles exhibited better activity in the cavitation-Fe_3_O_4_–H_2_O_2_ system than in its cavitation-less variant.

The synthesis method is one of the most critical factors influencing the properties of PVDF. In light of the growing interest in the piezoelectric characteristics of this material, promising results in enhancing its functional performance have been achieved through electrospinning technology, which promotes the formation of the β-phase and the development of highly oriented nanostructures.^17^ Furthermore, the incorporation of nanoparticles such as Fe_3_O_4_ has been shown to stabilize the β-phase, making PVDF/Fe_3_O_4_ composites particularly attractive for catalytic applications.^11,12^ In laboratory conditions, ultrasound is widely employed as a means of activating piezoelectric materials based on PVDF composites, including PVDF/Fe_3_O_4_.^18^ The mechanical stimulation induced by ultrasound triggers the piezoelectric effect through material deformation, which in turn enhances photocatalytic activity. At a certain intensity, ultrasound induces cavitation—the formation, growth, and collapse of microbubbles in a liquid medium.^19^ This phenomenon results in localized spikes in temperature and pressure, reaching extreme values (up to 5000 atm and 15,000 K), which can significantly intensify catalytic reactions.^20^

However, it is known that cavitation is strongly dependent on the applied frequency.^21^ In general, as the frequency of ultrasound increases, the bubble lifetime decreases and the size of cavitation bubbles decreases. In other words, the cavitation threshold increases with increasing frequency, and consequently, the cavitation intensity decreases, which leads to a decrease in the maximum temperature and pressure achieved at the bubble collapse.^22,23^

In this paper, the piezo-photocatalytic activity of PVDF/Fe_3_O_4_ nanofibers prepared by electrospinning was investigated. The main focus is to study the effect of ultrasonic frequency and different light sources on the decomposition efficiency of a model organic pollutant commonly known as methylene blue.

## 2 Materials and Methods

### 2.1 Synthesis

For the synthesis of pure PVDF samples, the electrospinning method was used to obtain thin fibers with diameters in the order of microns and nanometers. In this case, the basic material is PVDF (Sigma-Aldrich, 427144-100G) with a molecular weight of 275000 g/mol. A 20 wt % polymer solution was prepared in a mixture of dimethyl sulfoxide (DMSO) and acetone in a volume ratio of 7:3. Such a polymer solution allows achieving the desired viscosity for electrospinning, which is critical for the formation of quality fibers. The electrospinning process was conducted at a voltage of 50 kV at a solution flow rate of 20 μL/min^–1^. This ensured stable solution flow and uniform fiber formation. The distance between the needle tip and the rotating collector was set at 20 cm, allowing the fibers time to solidify before they reached the collector. The collector covered with aluminum foil was rotated at a speed of 2000 rpm, which promoted uniform distribution of fibers on the surface and formation of dense fiber mats. The processing time was 60 min. After completion of the process, the fibers were dried at room temperature overnight to remove the remaining solvent. The humidity level in the room was maintained at 42%, which prevented excessive moisture and ensured the stability of the resulting materials.

The synthesis of PVDF/Fe_3_O_4_ composite fibers was carried out according to a similar protocol using electrospinning. For this purpose, 10 wt % Fe_3_O_4_ nanopowder (Sigma-Aldrich 637106-25G, Particle size 50–100 nm) was added to PVDF solution. For the synthesis of composite fibers, a different concentration of PVDF solution, 16 wt %, was used to obtain a solution with the desired viscosity for electrospinning. The voltage for electrospinning remained the same (50 kV), but the solution delivery rate was increased to 65 μL/min^–1^ to account for the higher viscosity of the solution. The electrospinning time was increased to 90 min to ensure that enough high-density fibers were formed. The atmosphere humidity was reduced to 15% to prevent excessive moisture from disturbing the fiber formation process. The nanofibers were collected as nonwoven mats and dried at room temperature overnight to ensure a stable structure and the removal of residual solvents.

### 2.2 Measurement Methods

The morphology of the samples was studied on a LYRA3 scanning electron microscope (SEM) (Tescan, Brno, Czech Republic) with an X-Max 50 EDS detector (Oxford Instruments, Abingdon, UK) and Helios NanoLab 660 (ThermoFisher Scientific, Brno, Czech Republic). The samples were coated with 15 nm thick carbon using a Leica EM ACE600 coating device (Leica Microsystems, Wetzlar, Germany). The images of the synthesized materials obtained by SEM were analyzed by ImageJ software to calculate the fiber diameter distribution. FTIR measurements were performed using a Bruker (Billerica, Massachusetts, USA) with a resolution of 1 cm^–1^ and over 512 iterations.

X-ray photoelectron spectroscopy (XPS) was performed to determine the types of chemical bonds in the samples using an AXIS Supra instrument (Kratos Analytical Ltd., Manchester, UK), with results recorded at an emission current of 15 mA and a resolution of 20 for broad spectra and 80 for element-specific spectra. The resulting spectra were approximated using CasaXPS software (version 2.3.23, Kratos Analytical Ltd., Manchester, UK).

Raman spectra were acquired using a WITec alpha300 R system (WITec, Ulm, Germany) with a 532 nm excitation wavelength and a laser power of 1 mW. The signal was reconstructed from 50 accumulations, each with an integration time of 20 s, utilizing a 10x objective.

The magnetic properties of the composite were investigated using LakeShore 7400 vibromagnetometer (Lake Shore Cryotronics, Inc., Westerville, OH, USA) at room temperature.

The wettability of the samples was determined using the static contact angles based on the sessile droplet method using the Surface Energy Evaluation System device (See System E, Advex Instruments, Czech Republic). A 3 μL droplet of distilled water, glycol, ethylene glycol, and diiodomethane was manually dispensed on the sample surfaces, and the contact angle was determined by analysis of droplet images using See Software 7.0. Ten contact angle measurements were taken at 22 °C with relative humidity between 30% and 40%. The mean contact angle was calculated by averaging the static contact angles from the ten measurements.

### 2.3 Piezophotocatalytic Experiment

The experiment was conducted using methylene blue (MB) solution as a model pollutant. Membranes of 3 × 1 cm were immersed in a beaker (*V* = 50 mL) with dye solution (*V* = 20 mL, *C*_0_ = 2.5 mg/L). As a source of mechanical action, ultrasonic baths with a power of 240 W but with different frequencies were used: 40 kHz (FanYing Sonic, 10L, China) and 60/120 kHz (T-040TP, 10L, China). The location of the beaker in the ultrasonic bath, the type of beaker (GG-17), the depth of immersion in the bath (to the level of the solution inside the beaker) and the volume and temperature of the water in the bath were fixed in all experiments. A high-pressure mercury lamp without phosphor layer (Philips, 250 W) was used as the ultraviolet (UV) light source. The visible light source was a metal halide lamp (Osram, 75 W). The distance between the light source and the beaker was 10 cm. The temperature of the solution in the beaker was kept constant at 26 °C. Solution samples were taken every 15 min and analyzed on an SF-2000 spectrophotometer. The MB concentration was determined at a wavelength of 663.7 nm.

The following MB decomposition experiments were performed to study the influence of various factors:

- Irradiation with light in the presence of a catalyst (photocatalysis, PhC);
- Irradiation with light in the absence of a catalyst (photolysis, Ph);
- Exposure to ultrasound without a catalyst (sonolysis, S);
- Exposure to ultrasound with a catalyst (piezocatalysis, PC);
- Simultaneous exposure to light and ultrasound in the presence of a catalyst (piezo-photocatalysis, PPhC).

The degree of dye degradation was determined by calculating the ratio of the current dye concentration to the initial concentration.

Experiments with reactive oxygen species (ROS) scavengers were conducted under standard conditions by adding 1 mM of each scavenger to the reaction system. The reactive species h^+^, e^–^, ^•^OH and ^•^O_2_^–^ were selectively trapped using disodium ethylenediaminetetraacetate (EDTA), silver nitrate (AgNO_3_), *p*-benzoquinone (BQ), isopropyl alcohol (IPA).

For the analysis of ^•^OH fluorescence, the experiment was conducted under similar piezocatalytic conditions. Instead of MB solution, a solution of 3 mmol terephthalic acid was used, and the pH was adjusted to 10.0 using NaOH solution. The fluorescence spectra of the solution were recorded using a Hitachi F-4500 fluorescence spectrophotometer with an optical path length of 1.0 cm, excited at a wavelength of 315 nm, and the fluorescence emission peak at 430 nm was recorded at fixed time intervals.

The potential for H_2_O_2_ generation during the PPhC process was also investigated. Distilled water was used as the reaction medium. To determine the H_2_O_2_ yield during the PPC reaction, 4 mL of the reaction suspension was sampled after 45 min. After filtration, 500 μL of the filtrate was mixed with 50 μL of a 0.01 M solution of ammonium molybdate (H_32_Mo_7_N_6_O_28_) and 2 mL of a 0.1 M potassium iodide (KI) solution. After allowing the reaction to proceed for 15 min, the absorbance of the resulting mixture was recorded at 350 nm using a UV–vis spectrophotometer.

To quantify the generated piezoelectric potential, a 3 × 1 cm piezoelectric nanogenerator (PENG) was fabricated from the composite membrane. The PENG device features a sandwich-like structure, consisting of the 3 × 1 cm membrane sample placed between two copper tape electrodes, to which twisted copper current leads were soldered. The entire assembly was subjected to vacuum treatment to remove trapped air and subsequently encapsulated with a PET film to ensure mechanical stability and electrical insulation.

The aluminum foil erosion method was additionally employed for a qualitative assessment of the intensity of mechanical effects at different ultrasonic frequencies. A circular aluminum foil sheet with a diameter of 35 mm was placed in a beaker and exposed to ultrasound under conditions identical to those used in the main experiments. The exposure time was experimentally set to 2 min to ensure sufficient erosion of the foil.

Mechanical stimulation of the PENG was applied using an overhead mechanical stirrer operating at a fixed frequency of 4 Hz, providing periodic deformation of the membrane. The electrical output signals generated by the PENG in response to mechanical excitation were recorded using a UTD2102CL+ digital oscilloscope. The oscilloscope probes were directly connected to the output terminals of the PENG to capture voltage signals with high temporal resolution.

## 3 Results and Discussion

Figure 1 shows SEM images and histograms of nanofiber distribution of pure PVDF and PVDF/Fe_3_O_4_ composite. The pure PVDF membrane (Figure 1a) is characterized by micron diameter fibers with chaotically arranged and intertwined structure. The average diameter of the fibers is about 2 μm, which is confirmed by the distribution histogram. The enlarged image shows that the surface of the fibers is covered with large pores, indicating a developed microporous structure of the material. The porous structure of the fibers may be due to the peculiarities of the electrospinning process and is associated with phase separation during solvent evaporation. Such pores are often formed due to the interaction of the polymer solution with the surrounding atmosphere (high humidity of the medium) and conditions of thermodynamic instability, leading to segregation of components in the process of fiber curing.

**Figure 1:**
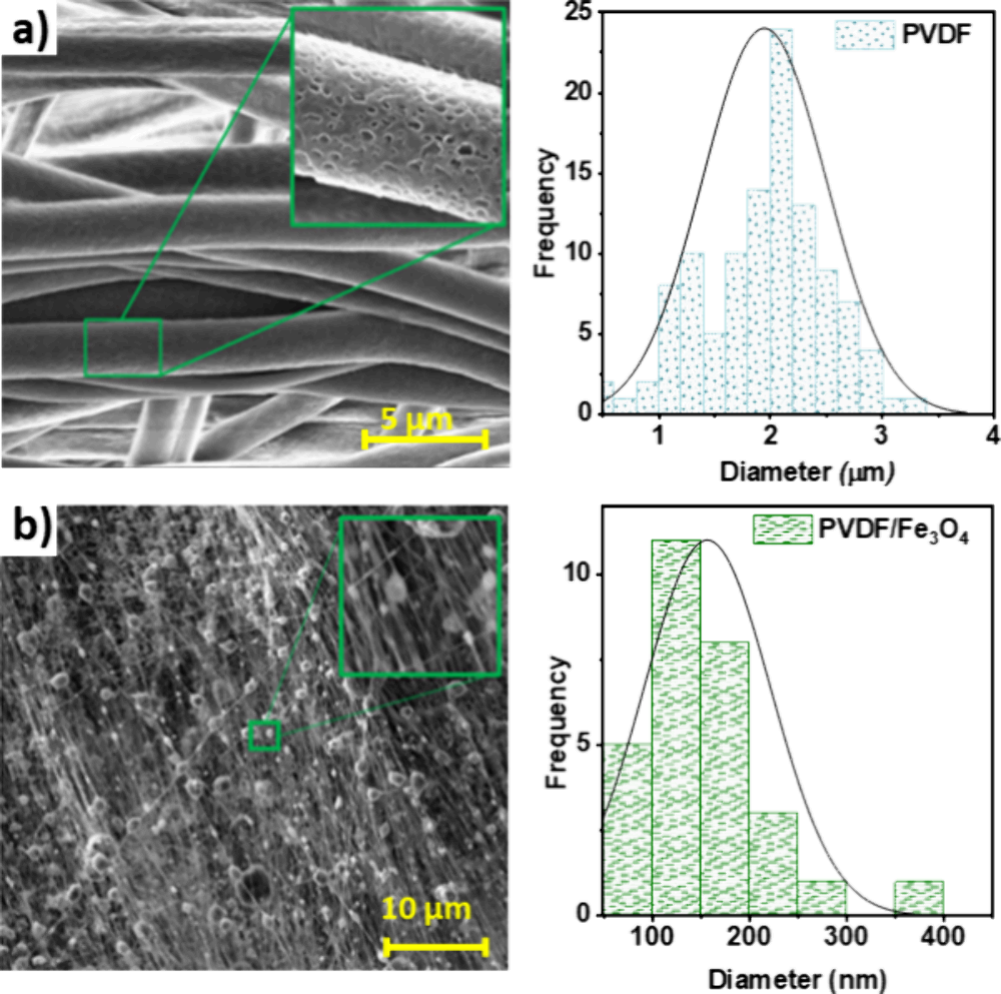
SEM images and histogram of fiber diameter distribution: (a) PVDF and (b) PVDF/Fe_3_O_4_.

The composite membrane shows a more complex morphology compared to pure PVDF (Figure 1b). The fibers are predominantly oriented, but agglomerates of magnetite nanoparticles (Fe_3_O_4_) are visible on their surface, which is clearly visible in the enlarged image fragment. These agglomerates increase the roughness of the surface and texturization. The agglomeration of Fe_3_O_4_ nanoparticles can be caused by their high specific surface area and interparticle magneto-dipole interactions. The average diameter of the fibers is about 150 nm, which is much smaller than that of a pure PVDF membrane. The decrease in fiber diameter can be attributed to the change in the rheological and electrophysical characteristics of the polymer solution during the electrospinning process. Although the electrical conductivity could not be measured correctly due to the small volume and high viscosity of the sample, its increase can be assumed by the addition of nanoparticles into the solution, which leads to an increase in the strength of the electric field acting on the Taylor cone and contributes to a more intense stretching of the polymer jet and, consequently, to a decrease in the diameter of the formed fibers. In addition, the introduction of nanoparticles affects the viscosity of the solution, facilitating the pulling of fibers. The reduction of fiber diameter in the composite membrane has a positive effect on its specific surface area and functional properties, especially in applications related to photocatalysis.

PVDF is known to have at least four crystalline phases α, β, γ and δ. The most interesting phase is the β-phase and to evaluate it, the phase distribution was quantitatively evaluated using FTIR total internal reflection spectroscopy. The obtained FTIR spectra of pure PVDF and PVDF/Fe_3_O_4_ composite membrane (Figure 2) allowed a detailed analysis of the crystalline phases present in the samples and an evaluation of the effect of the addition of magnetite nanoparticles on the phase composition of the polymeric material. Figure 2 shows the spectra in the range (400 to 1600) cm^–1^.

**Figure 2:**
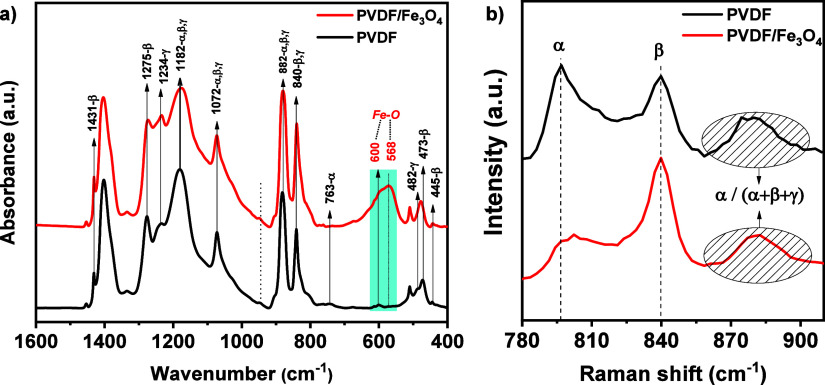
FTIR (a) and Raman (b) spectra of pure PVDF and PVDF/Fe_3_O_4_ fibers.

Both samples showed the presence of characteristic absorption bands for α- and β-phases. The peaks at 763 cm^–1^ and 1379 cm^–1^ confirm the presence of a nonpolar α-phase, which is consistent with previously published data.^24^ At the same time, intense absorption bands at 840 cm^–1^, 1275 cm^–1^, and 1431 cm^–1^ indicate the presence of piezoelectric β-phase, which is also in agreement with literature data.^24,25^

In the case of the composite membrane, the spectra acquire a more complex character. In addition to the absorption bands characteristic of PVDF, new bands at 568 cm^–1^ and 600 cm^–1^ are observed, which correspond to stretching and vibration of Fe–O bonds, respectively. These bands are a hallmark of the spinel structure of magnetite, confirming the successful introduction of Fe_3_O_4_ into the polymer framework.^26–28^

The absorption bands at 763 cm^–1^, 840 cm^–1^, characteristic of α-, β- and γ-phases, respectively, were used to estimate the content of the proportion of electroactive phase in the samples. The proportions of the phases were calculated using a technique based on the values of absorption coefficients and peak height differences (eq 1).

$$F_{\mathrm{EA}}=\frac{I_{\mathrm{EA}}}{\left(\frac{K_{840}}{K_{763}}\right)I_{763∼769}+I_{\mathrm{EA}}}\times 100\%$$ 1

where, *I*_EA_ and *I*_763∼769_ are absorption at ∼840* and 763∼769 cm^–1^, respectively; *K*_840*_ and *K*_763_ are absorption coefficients at the corresponding wave numbers, the values of which are 7.7 × 10^4^ and 6.1 × 10^4^ cm^2^ mol^–1^, respectively.

Figure 2b shows the Raman spectra in the region from 780 to 920 cm^–1^, which is chosen as representative because it shows the strongest contribution of all crystalline phases. The spectrum of pure PVDF is dominated by an intense peak at 797 cm^–1^, which corresponds to the α-phase. However, after the addition of magnetite, the intensity of this peak decreases markedly, indicating a decrease in the fraction of α-phase. At the same time, in the region around 840 cm^–1^, a significant enhancement of the peak associated with the β-phase is observed, indicating its dominant presence in the modified material.

Analysis of the phase composition calculated from the IR spectra data showed a significant difference in the phase ratio in the samples (Table 1).

**Table 1:** Calculated Phase Fractions for Pure PVDF and PVDF/Fe_3_O_4_ Fibers

**sample name**	**α-phase, %**	**F**_**EA**_ **, %**
PVDF/Fe_3_O_4_	0.2	99.8
PVDF	23.3	76.6

For pure PVDF, the content of α-phase is 23.3%, while the proportion of phases including β and γ phases (*F*_EA_ phases) is 76.6%. In the PVDF/Fe_3_O_4_ composite membrane, the α-phase is almost absent (less than 0.2%), while the proportion of *F*_EA_-phases reaches 99.8%, indicating significant changes in the crystalline structure of the material under the influence of magnetite nanoparticles. The formation of α-phase in pure PVDF may be due to the fact that during the electrospinning process the PVDF solution is subjected mainly to mechanical stretching and relatively low electric field, which leads to the formation of α-phase. The complete disappearance of α-phase in PVDF/Fe_3_O_4_, in our opinion, can be attributed to several factors. First, Fe_3_O_4_ nanoparticles can act as nucleation centers, promoting the formation of a more polar β-phase due to interaction with polar groups −CF_2_– and −CH_2_– in the polymer matrix leading to self-polarization.^29^ Second, an increase in the electrical conductivity of the solution leads to an increase in the electric field strength during the electrospinning process, promoting additional reorientation of PVDF molecules along the electric field line.^30^ In addition, increasing the electric field strength can also prevent the relaxation of the polymer chains into the α-configuration, further promoting the dominance of the β-phase.

The samples were examined by X-ray photoelectron spectroscopy (XPS) to evaluate changes in surface chemistry and interactions between the polymer matrix and nanoparticles. Analysis of the C 1s and F 1s spectra revealed how the surface chemistry changes when magnetite is added to PVDF (Figure 3).

**Figure 3:**
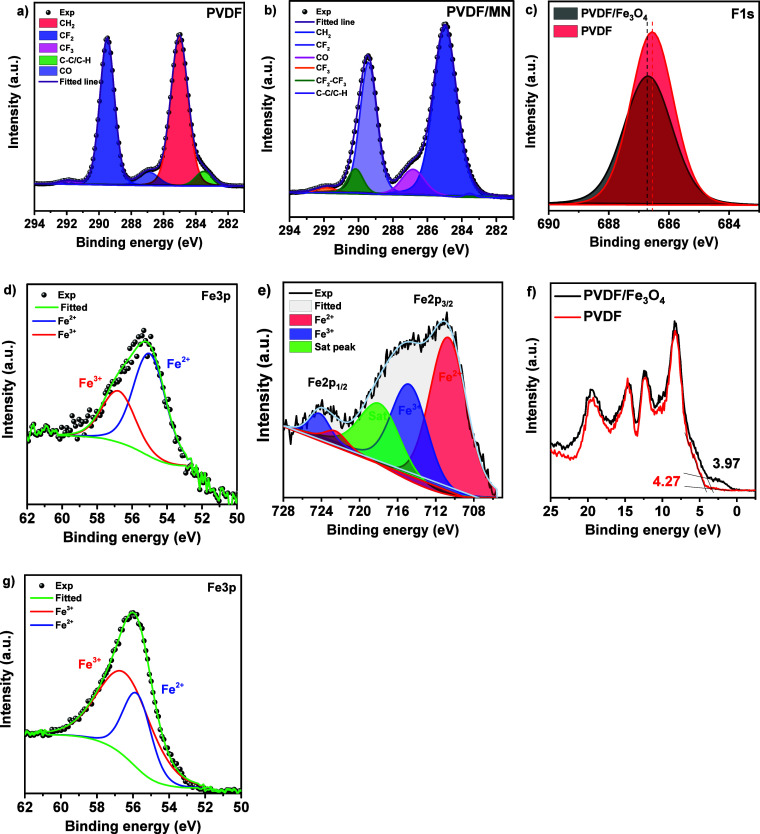
XPS spectra: (a, b) C 1s PVDF and PVDF/Fe_3_O_4_ nanofibers, (c) F 1s PVDF and PVDF/Fe_3_O_4_ nanofibers, (d) Fe 3p PVDF/Fe_3_O_4_, (e) Fe 2p PVDF/Fe_3_O_4_, (f) VB PVDF and PVDF/Fe_3_O_4_, and (g) Fe 3p Fe_3_O_4_.

The C 1s spectrum of pure PVDF (Figure 3a) after treatment by Gaussian approximation shows several characteristic peaks. The main peaks located at 289.5 and 285.0 eV characterize the carbon in the CF_2_ and CH_2_ groups, respectively. The presence of signal at 286.8 eV indicates the presence of C–O groups on the surface, while the peak at 283.5 eV corresponds to C–C/C–H bonds. In addition, the peak at 291.9 eV indicates the presence of CF_2_–CF_3_ group.^31^ This specific group is very rarely mentioned in the literature, but the base CF_2_ and CF_3_ groups are commonly discussed,^32–35^ and are in the close proximity to the 291.9 eV peak, so their combination is expected to be located in its vicinity. The ratio of the intensities of the CF_2_ and CH_2_ peaks indicates a uniform distribution of groups on the surface of pure PVDF. In the case of PVDF/Fe_3_O_4_ composite (Figure 3b), an increase in the intensity of the CH_2_ peak and a decrease in the intensity of CF_2_ are observed in the C 1s spectrum. This phenomenon is explained by the ion-dipole interaction between the magnetite surface and CF_2_ dipoles of PVDF. The positively charged Fe_3_O_4_ surface interacts with the negatively charged CF_2_ dipoles, which causes structural changes in PVDF and leads to its additional self-polarization. These changes contribute to the orientation of the polymer chains and increase the β-phase content. Analysis of the F 1s spectrum (Figure 3c) for pure PVDF reveals a symmetric peak around 686.5 eV associated with fluorine in CF_2_ groups. However, in the PVDF/Fe_3_O_4_ composite, the F 1s peak shifts to the higher energy region and acquires asymmetry, which confirms the presence of ion-dipole interaction between CF_2_ groups and the magnetite surface.^18^

The stoichiometric Fe_3_O_4_ can be represented as FeO· Fe_2_O_3_, hence the ratio of Fe^2+^:Fe^3+^ should be 1:2. The Fe 3p region (Figure 3d) was used for quantitative analysis of Fe^3+^ and Fe^2+^ because it is represented by a single peak with no interfering satellite signals for each chemical state. Based on the integral areas of the peaks obtained from the deconvolution of the Fe 3p spectrum of the nanoparticles (Figure 3g), the Fe^2+^: Fe^3+^ ratio for pure magnetite was found to be 1:2, which corresponds to its stoichiometry. In the case of PVDF/Fe_3_O_4_ composite, a dramatic change is observed and the Fe^2+^:Fe^3+^ ratio reaches 2:1. The study presented in^36^ highlights the significant effect of dimethyl sulfoxide (DMSO) on the properties of Fe_3_O_4_ synthesized by coprecipitation. It is found that DMSO plays the role of a stabilizer that changes the morphology and size of nanoparticles through active interaction with their surface. This interaction can lead to the partial reduction of Fe^3+^ to Fe^2+^, especially with the local change in the electrochemical potential caused by the interaction of Fe^3+^ with −CF_2_– dipoles in PVDF. This effect requires further investigations. Analysis of the Fe 2p region (Figure 3e) for the PVDF/Fe_3_O_4_ composite showed the presence of five peaks corresponding to Fe^3+^ and Fe^2+^ doublets, as well as a satellite peak.^37–39^ The valence band spectrum (Figure 3f) was investigated to evaluate the electronic structure of the materials. For the PVDF/Fe_3_O_4_ composite, changes in the valence band spectrum are observed: in the range of 0–4 eV, an increase in the density of states is recorded, indicating the appearance of additional energy levels. This phenomenon is related to the interfacial interaction between the nanoparticle surface and the PVDF polymer matrix. Such interaction leads to structural ordering of the material and formation of long-lived metastable states.^18^ The position of the valence band for the composite is shifted by 0.3 eV.

Measurement of the marginal contact angle (MCA) of water wetting is an important method that can confirm the conclusions obtained using XPS. This method allows us to evaluate changes in the chemical composition and surface polarity revealed by spectroscopic analysis through their effect on the hydrophilicity or hydrophobicity of the material.^40^

Measurement of the wetting angle (Figure 4) showed a significant decrease in the value for the PVDF/Fe_3_O_4_ composite compared to pure PVDF: 145.2° and 81.8° indicating the transition of the material from the hydrophobic category to the moderately hydrophilic one. The high wetting angle for pure PVDF is due to the presence of CF_2_-groups, which are characterized by pronounced hydrophobic properties due to the high electronegativity of fluorine atoms.^41^ The decrease in the fraction of CF_2_ groups recorded in the C 1s and F 1s spectra and the increase in the concentration of polar groups such as C–O directly correlate with the increase in wettability.

**Figure 4:**
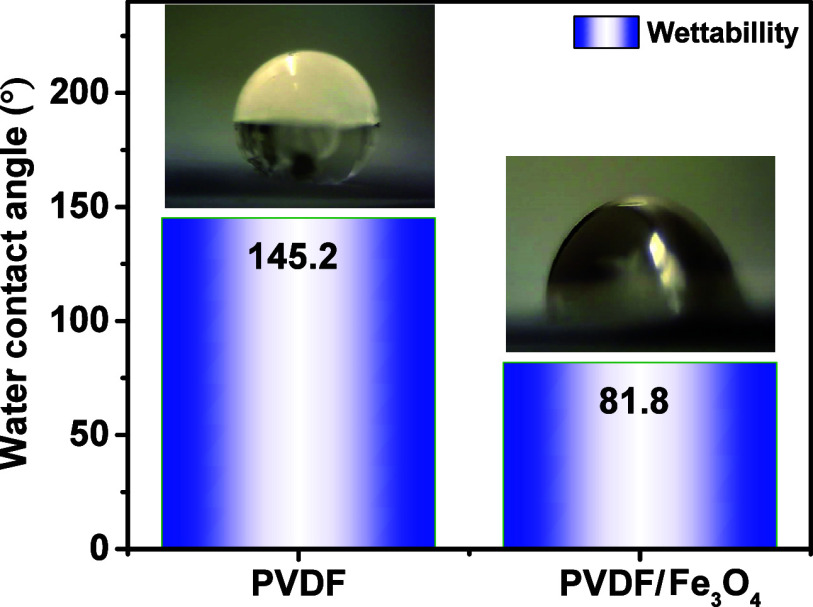
Water wetting edge contact angle for PVDF and PVDF/Fe_3_O_4_ and droplet images.

These results are of key importance for the application of the material in piezo-photocatalysis processes. The high hydrophilicity provides better wetting of the surface by aqueous solutions, which promotes more efficient contact between the catalyst active centers and reagent molecules. In addition, the increased surface polarity can improve the adsorption of polar organic pollutants or water, which is critical for contaminant degradation processes under light and mechanical stress.

Based on the results obtained, these materials were further applied in piezo-stimulated photocatalysis processes for the degradation of methylene blue. This model system has been widely used to evaluate the effectiveness of catalysts in removing organic pollutants from aquatic environments. Combining the piezoelectric properties of PVDF with the catalytic activity of magnetite allowed us to study the synergistic effect of their interaction under the influence of mechanical and light stimuli.

Figure 5 shows the results of methylene blue (MB) degradation study under different treatment conditions using PVDF/Fe_3_O_4_ composite. Blank experiments showed that when exposed to UV visible light (Photolysis (Ph)), the degradation rate of the dye reached 69% in 60 min. Under ultrasonic treatment (sonolysis (S)) at 40, 60, and 120 kHz, the degradation efficiency was 66%, 24%, and 10%, respectively. Photocatalysis (PhC) showed a degradation degree of 57%, indicating that the composite has no significant photocatalytic properties.^42^ This behavior is due to the physical and chemical features of the composite. First, the structure of PVDF fibers promotes the scattering of light radiation, which limits the interaction of light with Fe_3_O_4_ nanoparticles and reduces the generation of active electron–hole pairs.^43^ Second, the high recombination rate of these pairs additionally prevents the formation of highly active oxygen forms necessary for photocatalytic processes.^44^

**Figure 5:**
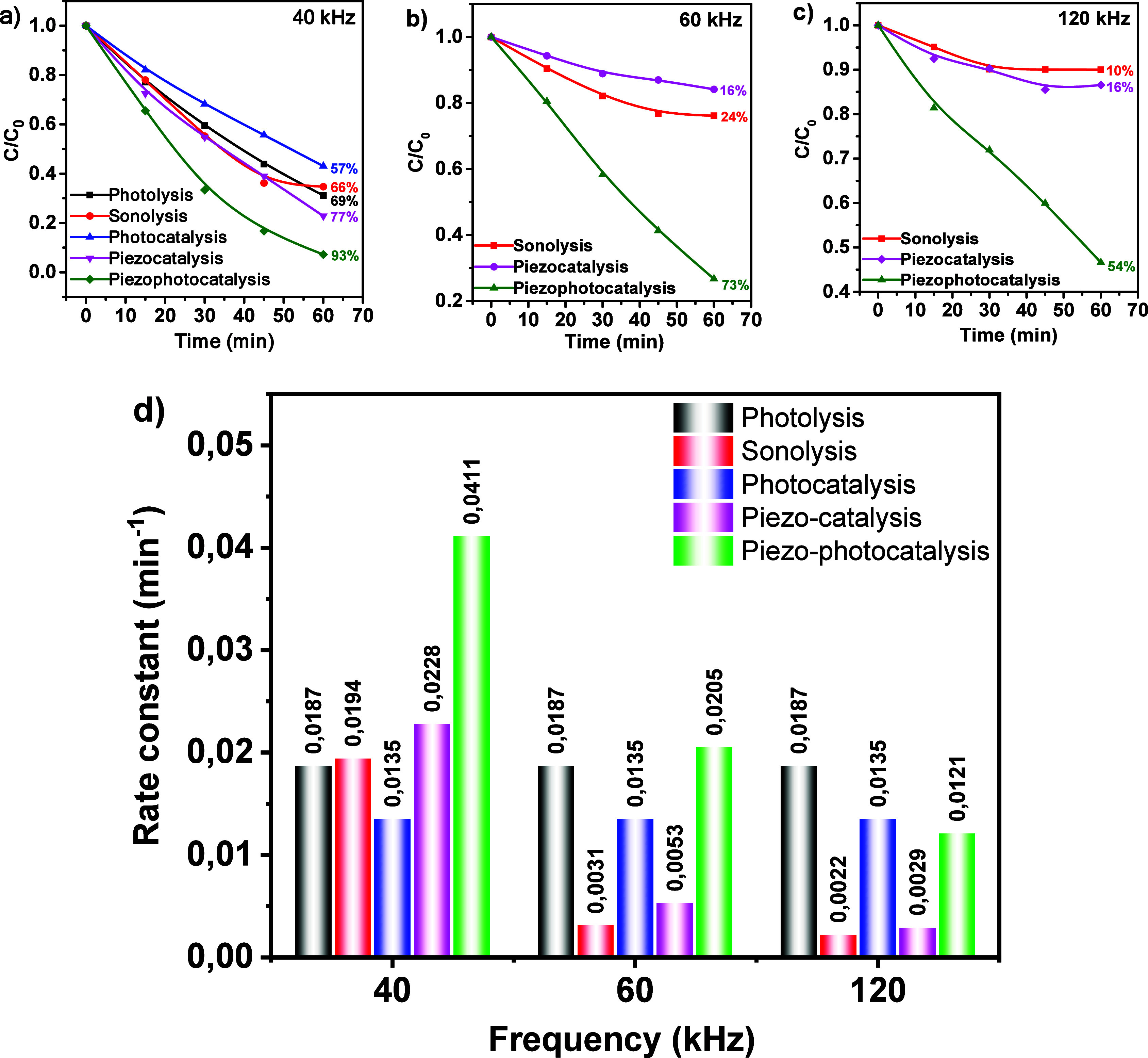
MB decomposition curves in different processes under UV–visible light irradiation and ultrasonic exposure with frequencies of (a) 40, (b) 60, and (c) 120 kHz. (d) Values of rate constants for all processes.

The results of piezocatalysis (PC) performed in the dark showed significantly higher dye degradation efficiency. At 40 kHz, the degradation rate was 77%, which indicates the generation of piezoelectric potential during mechanical deformation of the composite. Under the influence of ultrasonic cavitation, piezopolarization phenomena occur in the material, leading to the induction of electric charges on the PVDF surface. This polarization electric field promotes the initiation of redox reactions leading to the formation of highly active forms of oxygen, such as hydroxyl radicals (·OH), contributing to the effective degradation of the dye.

The results of the experimental study of MB decomposition using piezocatalysis at different ultrasonic frequencies show the dependence of decomposition efficiency on the characteristics of acoustic cavitation. At a frequency of 40 kHz, a noticeable increase in the decomposition efficiency is observed compared to the control sonolysis. This is attributed to the dominance of transient cavitation, in which there is an intense growth of cavitation bubbles to a critical size and their subsequent explosion. These microexplosions create extreme localized conditions (high temperatures and pressures) accompanied by the generation of significant amounts of active radicals such as hydroxyl radicals (^•^OH). These processes also induce mechanical effects on the composite, activating its piezocatalytic properties, which significantly increases the efficiency of MB decomposition. When the ultrasound frequency is increased up to 60 kHz, the intensity of transient cavitation decreases. Bubbles under these conditions have less time to grow to a critical size, which reduces their explosive activity. This leads to a decrease in temperature, pressure and consequently in the number of radicals produced. This is contrary to some published results,^45^ where the influence of frequency on the cavitation bubble lifetime is the opposite. This might be caused by the multiparametric nature of the issue, as the bubble lifetime is influenced by the thickness of the sample, its surface, ratio of piezo- to triboelectric effect, phase concentration of the sample etc. This issue requires in-depth simulation and more detailed research to present a confident conclusion.

Reducing the mechanical intensity of ultrasound also limits the activation of the piezoelectric properties of the composite, which explains the minimal difference in degradation results compared to the control sonolysis experiment. At 120 kHz, stable cavitation prevails, where bubbles do not reach a critical size and simply oscillate. These conditions do not create extreme temperatures and pressures, and the generation of active radicals is limited. The microstirring of the medium that occurs in stable cavitation has no significant effect on the mechanical activation of the composite, which explains the low efficiency of MB decomposition at this frequency. Thus, the experimental results confirm that the efficiency of piezocatalytic dye decomposition is determined by the degree of interaction between the mechanisms of transient cavitation and piezocatalysis. At a frequency of 40 kHz, an optimal combination of conditions for these processes is observed: intense microbursts of bubbles provide a high generation of active radicals and effective mechanical impact on the composite. At higher frequencies (60 and 120 kHz), the weakening of transient cavitation and the predominance of stable cavitation significantly limit the synergistic interaction between cavitation and piezocatalysis, which reduces the efficiency of the process. These findings emphasize the importance of selecting the optimal ultrasound frequency to maximize the degradation efficiency of organic pollutants.

The sample exhibits high piezo-photocatalytic activity (PPhC) - 93% of the dye was degraded in 60 min when exposed to 40 kHz ultrasound. Under the action of ultrasound in the polymer matrix of PVDF an electric field is created, which effectively separates charge carriers (electrons and holes), preventing their recombination. This promotes the active participation of these carriers in redox reactions, leading to effective decomposition of the pollutants. However, with increasing ultrasonic frequency, a decrease in the efficiency of the process is observed. At 60 kHz, the degree of MB decomposition is 73%, while at 120 kHz, it is only 54% (Figure 5). These changes can be explained by the decrease in the intensity of transient cavitation, which reduces the number of active radicals and thus the decomposition efficiency and correlates with the results of piezocatalysis. To analyze the decomposition kinetics in more detail, the reaction rate constants were calculated using the pseudo-first-order Langmuir–Hinshelwood model. The rate constants for all processes are plotted in the diagram in Figure 5d. The rate constant for photocatalysis (*k*_PhC_ = 0.0135 min^–1^) is lower than the value for photolysis (*k*_Ph_ = 0.0187 min^–1^). This unexpected decrease in photocatalytic activity relative to photolysis can be explained both by the shielding of light by the catalyst due to absorption, reflection, and scattering of part of the light, reducing the amount of energy available for direct photolysis of MB molecules, and by the recombination of electrons and holes in the photocatalyst. Piezocatalytic activity significantly outperforms sonolysis at all frequencies, with the largest relative effect observed at 60 kHz and the largest absolute effect at 40 kHz. Based on this, to evaluate the presence or absence of synergistic effect in the PPhC process, it is necessary to consider the contributions of PhC and PC. The results of the analysis are presented in Table 2.

**Table 2:** Data of Rate Constants for Different Decomposition Processes of MB at Different Frequencies

frequency (kHz)	PhC (k_PhC_, min^–1^)	PC (k_PC_, min^–1^)	PPhC (k_PPhC_, min^–1^)	sum (k_PhC_+k_PC_, min^–1^)	excess of k_PPhC_ over the sum, %	synergistic effect
40	0.0135	0.0228	0.0411	0.0363	13	yes
60	0.0135	0.0053	0.0205	0.0188	9	yes
120	0.0135	0.0029	0.0121	0.0164	–26	no

The synergetic effect in piezo-photocatalysis is most pronounced at 40 kHz, where the maximum excess of *k*_PPhC_ over the sum of the contributions of photocatalysis and piezocatalysis (by 13%) is observed. At this frequency, ultrasonic waves provide high cavitation energy and light additionally stimulates catalyst activation. The combination of these factors enhances the formation of active radicals (^•^OH, ^•^O_2_^–^) and leads to a pronounced synergistic effect.^46,47^ At 60 kHz, the synergistic effect persists but becomes less pronounced (9%), and at 120 kHz there is no synergistic effect due to the weakening of the ultrasonic effect.

Considering the impracticality of using UV light and the bandgap width of magnetite nanoparticles, similar experiments on MB decomposition under visible light irradiation were performed (Figure 6).

**Figure 6:**
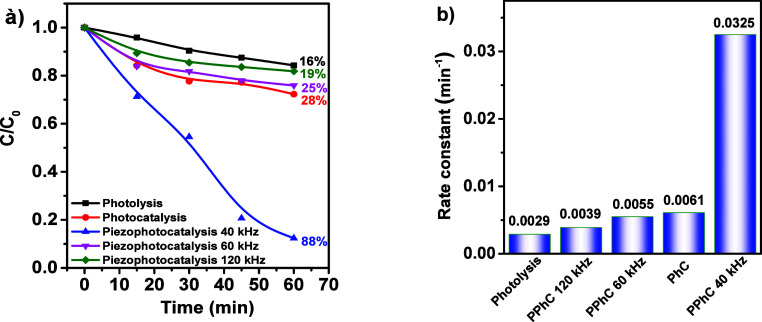
(a) Piezophotocatalytic MB decomposition curves at different frequencies and visible light irradiation and (b) kinetic rate constants.

To identify the contribution of MB self-decomposition, photolysis was repeated, with an efficiency of 16%. MB decomposition by photocatalysis showed an efficiency of 28%. The maximum efficiency of PPhC was achieved at an ultrasound frequency of 40 kHz, amounting to an impressive 88%. Interestingly, when exposed to visible light, the efficiency of PPhC remained comparable to the results obtained under UV–visible radiation. However, as with UV irradiation, increasing the ultrasound frequency significantly decreases the process efficiency: at 60 kHz it drops to 25%, and at 120 kHz it drops to 19%. The rate constants were 0.0029, 0.0061, 0.0325, 0.0055, and 0.0039 min^–1^ for Ph, PhC, PPhC at 40 kHz, PPhC at 60 kHz and PPhC at 120 kHz, respectively.

Thus, the obtained results clearly demonstrate that the efficiency of piezo-assisted photocatalysis significantly increases at low ultrasound frequencies. According to the literature, the generation of sonochemical radicals is known to be maximized at ultrasonic frequencies above 100 kHz.^48^ Therefore, the observed effect at lower frequencies can be primarily attributed to the mechanical nature of ultrasonic stimulation. Specifically, as reported in ref 49_,_ cavitation bubbles formed at frequencies below 100 kHz are able to grow over longer acoustic cycles. This results in more violent collapses of the bubbles, which, while generating fewer chemical effects (such as radical formation), enhance mechanical phenomena such as shock waves, microjetting, and asymmetric bubble collapse near solid surfaces. Consequently, it can be assumed that these mechanical effects play a decisive role in the activation of the piezoelectric response and, subsequently, in initiating the photocatalytic process.

To qualitatively assess the intensity of mechanical action at different ultrasonic frequencies used in this study, acoustic field visualization was performed by immersing aluminum foil in the working media of the experimental setups. The results are presented in Figure 7.

**Figure 7:**
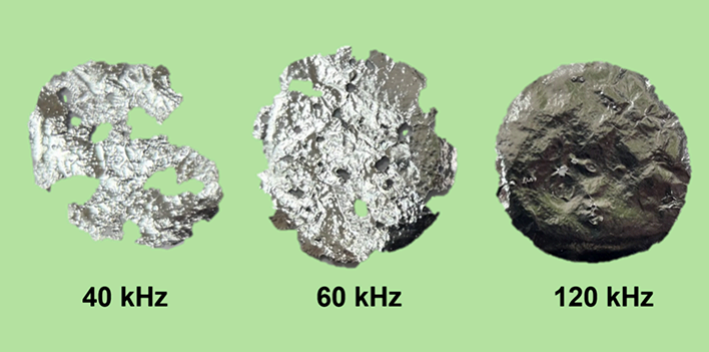
Longitudinal view of aluminum foil erosion experiments (exposure time: 2 min).

At low ultrasonic frequencies (40/60 kHz), the aluminum foil exhibited substantial erosion, indicating strong mechanical effects induced by acoustic cavitation (Figure 6a). The most pronounced damage was observed in the system operating at 40 kHz, where the foil surface experienced the highest degree of degradation. In contrast, in the setup operating at 120 kHz, no visible signs of erosion were observed, clearly suggesting that mechanical effects were significantly reduced at higher frequencies.

These findings establish a direct correlation between the intensity of mechanical action in the treated medium and the relative contribution of the piezocatalytic mechanism to methylene blue degradation. This highlights the critical role of mechanically induced effects under ultrasonic fields in enabling efficient piezo-assisted photocatalysis across different frequency regimes.

In order to identify the primary active species involved in the decomposition of methylene blue (MB), trapping experiments were carried out under piezophotocatalytic conditions using various scavengers at ultrasonic frequencies of 40 and 60 kHz, with PVDF/Fe_3_O_4_ employed as the catalyst. This system was selected for further investigation due to its high catalytic efficiency. To selectively quench specific reactive species, the following scavengers were used: ethylenediaminetetraacetic acid disodium salt (EDTA) for holes (h^+^), silver nitrate for electrons (e^–^), p-benzoquinone (BQ) for superoxide radicals (^•^O_2_^–^), and isopropanol (IPA) for hydroxyl radicals (^•^OH). The results are shown in Figure 8. As can be seen, at both frequencies (40 and 60 kHz), hydroxyl radicals play a dominant role in the degradation of MB, as the addition of IPA almost completely suppresses the catalytic activity. Nevertheless, the generation of superoxide radicals is also observed in the system at both frequencies.

**Figure 8:**
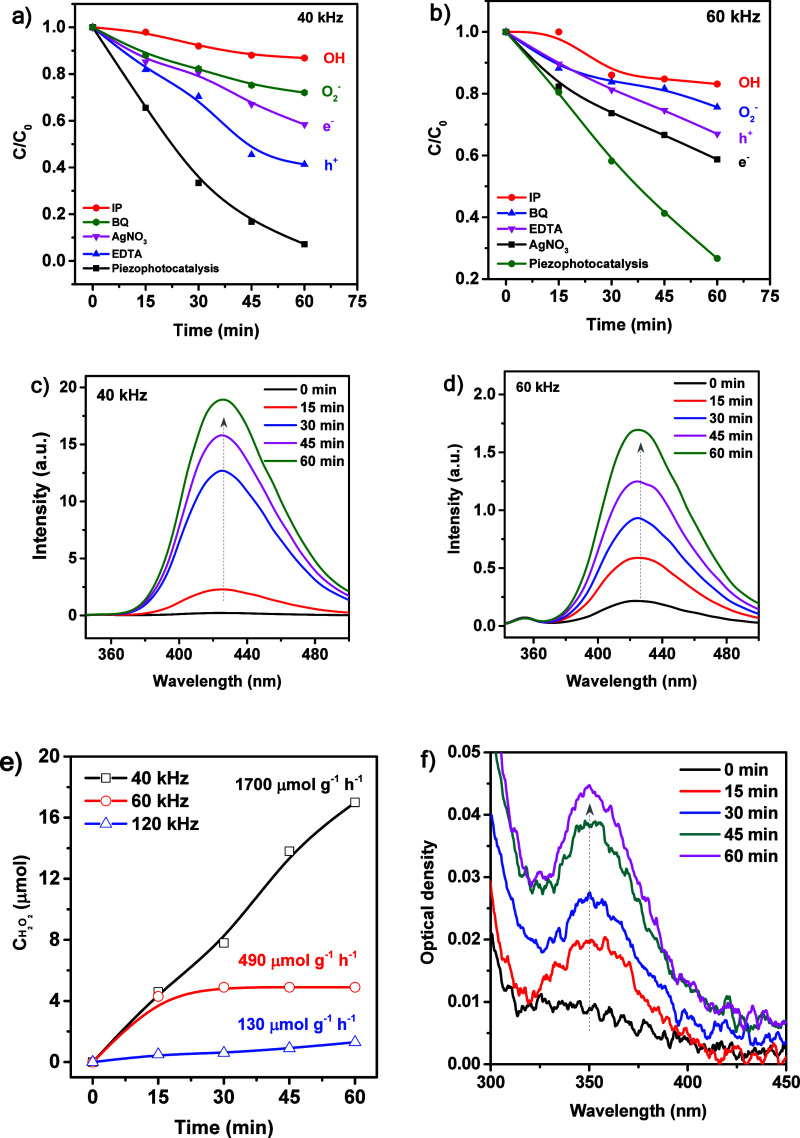
Piezophotocatalytic degradation of methylene blue (MB) using PVDF/Fe_3_O_4_ in the presence of different scavengers: (a) degradation curves at 40 and (b) 60 kHz; (c, d) fluorescence spectra of 2-hydroxyterephthalic acid for ^•^OH detection at 40 and 60 kHz, respectively; (e) piezophotocatalytic H_2_O_2_ generation profiles at 40, 60, and 120 kHz; (f) UV–vis absorption spectra confirming H_2_O_2_ generation under 40 kHz piezophotocatalysis.

To detect the presence of reactive species during the dye photodegradation process, fluorescence probing of ^•^OH was performed using terephthalic acid as a molecular probe (Figure 8c,d). The absence of a characteristic fluorescence peak at the beginning of the experiment (Figure 8c) indicates that ^•^OH species are not present in the initial reaction solution. However, upon exposure to ultrasonic vibration and light irradiation, the intensity of the emission peak at 430 nm gradually increases, attributed to the formation of the fluorescent product 2-hydroxyterephthalic acid. Notably, the relative concentration of hydroxyl radicals, as estimated from the fluorescence intensity, is significantly higher—by more than an order of magnitude—at 40 kHz compared to 60 kHz.

The influence of ultrasonic frequency on the catalytic performance in piezophotocatalytic H_2_O_2_ generation was investigated under identical reaction conditions (10 mg catalyst, 60 min reaction time, constant light irradiation) (Figure 8e,f). The productivity of the catalyst was determined at three different ultrasonic frequencies: 40, 60, and 120 kHz. At 40 kHz, the H_2_O_2_ yield reached 17 μmol, corresponding to a productivity of 1700 μmol·g^–1^·h^–1^. When the frequency was increased to 60 kHz, the H_2_O_2_ yield decreased to 4.9 μmol, resulting in a productivity of 490 μmol·g^–1^·h^–1^. A further increase to 120 kHz led to a significantly lower H_2_O_2_ yield of only 1.3 μmol, with a corresponding productivity of 130 μmol·g^–1^·h^–1^. These results indicate that lower ultrasonic frequencies are more effective in promoting piezophotocatalytic H_2_O_2_ production under the applied conditions, likely due to stronger mechanical deformation and more efficient piezoelectric activation of the catalyst. Importantly, the productivity achieved at 40 kHz is notably high compared to values reported in the literature, particularly considering that the reaction was carried out in pure water without the use of sacrificial agents, which are commonly employed to enhance charge separation and H_2_O_2_ stability.^50^ The high efficiency under such minimalistic conditions highlights the strong synergistic effect of light and piezo-induced charge modulation in the system, offering a promising strategy for sustainable and additive-free H_2_O_2_ production. For illustrative purposes, Figure 8f presents the UV–Vis spectra of H_2_O_2_ generation during the PPhC process at 40 kHz.

The light-to-chemical conversion (LCC) efficiency was calculated to evaluate the effectiveness of the piezophotocatalytic process at 40 kHz in converting light energy into chemical energy stored in H_2_O_2_.^51,52^ The Gibbs free energy change for H_2_O_2_ formation from water was taken as 117 kJ mol^–1^. In the experiment, 17 μmol of H_2_O_2_ was produced under light irradiation with an overall irradiance of 70 mW cm^–2^ over an effective area of 3 cm^2^ for 60 min. The total light power input was therefore 0.21 W, resulting in a total energy input of 756 J over the course of the reaction. The chemical energy stored in the produced H_2_O_2_ was calculated to be 1.989 J. Ignoring the contribution of ultrasonic power—since its role is primarily to modulate charge separation rather than directly supply energy—the LCC efficiency was calculated using the equation:

$$\mathrm{LCC}\;\mathrm{efficiency}\;(\%)=\frac{\Delta G_{H_{2}O_{2}}\times n_{H_{2}O_{2}}}{P_{\mathrm{light}}\times t}\times 100\%$$ 2

Substituting the values yielded an LCC efficiency of approximately 0.26%, indicating the proportion of incident light energy effectively converted into chemical energy under the given conditions.

The potential of magnetoelectric catalysis is of particular interest. In this context, an experiment was conducted on the degradation of MB in an alternating magnetic field using the PVDF/Fe_3_O_4_ composite. Figure 9a shows the digital model of the alternating magnetic field source assembled using permanent magnets. The device is capable of generating a magnetic field with a frequency of up to 2 Hz and an amplitude of 0.3 T at low rotational speeds.^53^ The M–H hysteresis loops for the PVDF/Fe_3_O_4_ composite at 300 K (Figure 9b) display typical behavior characteristic of Fe_3_O_4_-based materials.^54,55^ Catalytic experiments were performed at a magnetic field frequency of 1.3 Hz. In the absence of a magnetic field (red curve in Figure 9c), only weak adsorption of MB was observed. Upon application of the magnetic field, the degradation of MB reached 50% within 240 min. The proximity of the material to its magnetic saturation point facilitates the alignment of magnetic domains and the onset of magnetoelectric interactions, leading to membrane polarization and the generation of a piezopotential—essential for catalytic activity. These findings indicate that magnetoelectric coupling significantly accelerates catalytic processes, enhancing their efficiency and opening up promising opportunities for the application of such materials in water purification, waste remediation, and energy-related technologies.

**Figure 9:**
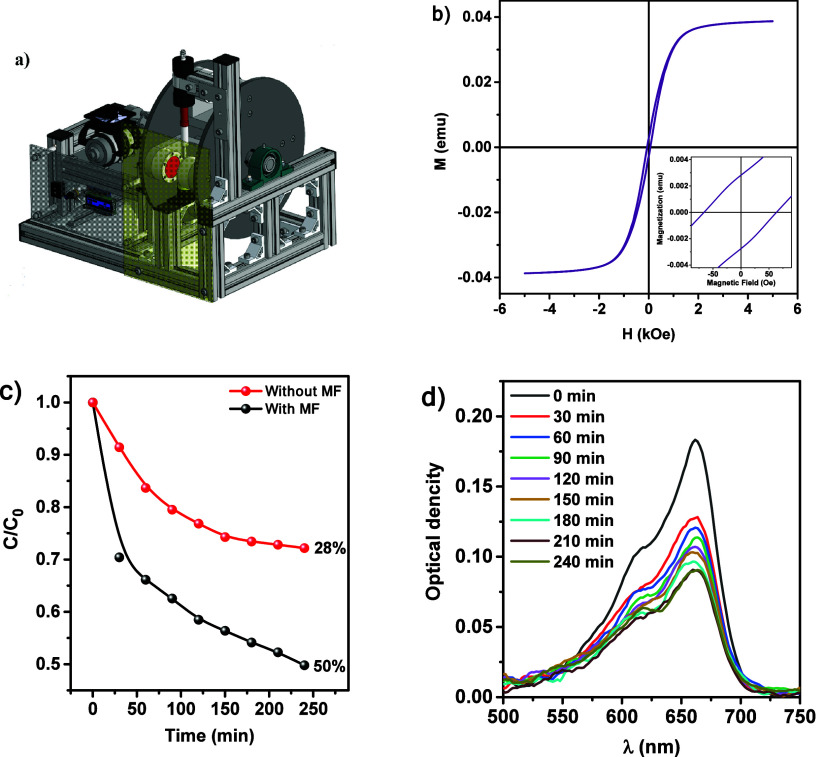
(a) Digital model of the alternating magnetic field (MF) source based on permanent magnets. (b) M–H hysteresis loops of the PVDF/Fe_3_O_4_ nanocomposite at 300 K. (c) Time-dependent degradation curves of methylene blue (MB) for the PVDF/Fe_3_O_4_ composite (1 mg·L^–1^ in 10 mL solution) with and without applied magnetic field. (d) UV–vis absorption spectra of MB solution during the catalytic experiment.

To experimentally confirm the generation of piezopotential under cyclic mechanical stimulation, a piezoelectric nanogenerator (PENG) was assembled. Figure 10 presents the time-dependent open-circuit voltage response of the PENG when actuated by an overhead stirrer operating at a fixed rotation frequency. The average amplitude of the generated voltage reaches approximately 2 V, confirming the effective piezoelectric activation of the material under mechanical excitation. An enlarged view highlights a single voltage pulse generated by the impact of a single blade stroke from the overhead stirrer. It can be observed that, following the initial peak in the output signal—attributed to the rapid mechanical deformation of the flexible piezoelectric material—the potential does not immediately return to zero. Instead, it exhibits a series of damped oscillations, which result from the inherent elasticity of the composite structure and the continued relaxation of internal mechanical stresses after the external force is removed. This transient behavior is characteristic of flexible piezoelectric systems, where the deformation-induced dipole realignment gradually decays over time. The decay time constant is on the order of 0.01 s, indicating rapid dissipation of mechanical energy and efficient electromechanical energy conversion in the system.

**Figure 10:**
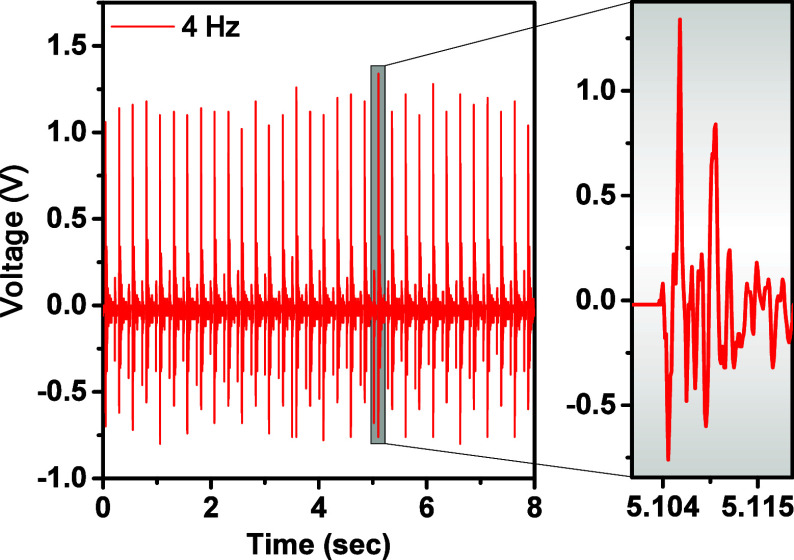
Time-dependent output voltages of nanocomposite PVDF/Fe_3_O_4_ at loading frequency of 4 Hz.

## 4 Conclusions

In this work, we demonstrated the fabrication and multifunctional catalytic performance of electrospun PVDF/Fe_3_O_4_ nanofibers. The electrospinning process, optimized for solvent composition, flow rate, and ambient humidity, enabled the formation of uniform nanofibers with an average diameter of ∼150 nm. Structural characterization revealed a drastic increase in electroactive β-phase content (up to 99.8%) upon Fe_3_O_4_ incorporation, confirmed by FTIR, Raman, and XPS. These structural modifications enhanced the piezoelectric properties and surface wettability of the composite, making it suitable for catalytic applications in aqueous environments.

The composite membrane was capable of generating piezoelectric voltage pulses (∼2 V) under low-frequency mechanical excitation (4 Hz), as demonstrated by PENG measurements. Under piezo-photocatalytic conditions, the system exhibited maximum MB degradation efficiency at 40 kHz (93% in 60 min), with a synergistic enhancement of 13% over the sum of individual piezocatalytic and photocatalytic effects. This frequency provided optimal cavitation intensity for mechanical activation of the piezoelectric response.

Scavenger and fluorescence experiments confirmed that hydroxyl radicals were the predominant reactive species. H_2_O_2_ generation was also highly efficient at 40 kHz, with a maximum yield of 17 μmol (1700 μmol·g^–1^·h^–1^), exceeding literature values under similar conditions and without sacrificial agents. The light-to-chemical conversion efficiency was calculated as 0.26%, demonstrating effective use of incident light energy.

Moreover, the composite was active under magnetically induced catalytic conditions: in an alternating magnetic field (1.3 Hz, 0.3 T), MB degradation reached 50% after 240 min. This indicates the potential of magnetoelectric coupling in the material, enabling charge generation through domain reorientation and mechanical stress induced by magnetic interactions.

Altogether, this study highlights the PVDF/Fe_3_O_4_ composite as a highly functional, flexible platform for environmentally friendly catalysis, with potential applications in piezocatalysis, magnetoelectric catalysis, and sustainable H_2_O_2_ production in clean water systems.
